# Association of *FOXD1* variants with adverse pregnancy outcomes in mice and humans

**DOI:** 10.1098/rsob.160109

**Published:** 2016-10-19

**Authors:** Paul Laissue, Besma Lakhal, Magalie Vatin, Frank Batista, Gaëtan Burgio, Eric Mercier, Esther Dos Santos, Christophe Buffat, Diana Carolina Sierra-Diaz, Gilles Renault, Xavier Montagutelli, Jane Salmon, Philippe Monget, Reiner A. Veitia, Céline Méhats, Marc Fellous, Jean-Christophe Gris, Julie Cocquet, Daniel Vaiman

**Affiliations:** 1 Institut Cochin, Université Paris Descartes, CNRS (UMR 8104), Paris, France; 2 Inserm, U1016 Paris, France; 3 Centro de Investigación en Genética y Genómica-CIGGUR, Grupo GENIUROS, Escuela de Medicina y Ciencias de la Salud, Universidad del Rosario, Bogotá, Colombia; 4 Department of Cytogenetics and Reproductive Biology, Farhat Hached University Teaching Hospital, Sousse, Tunisia; 5 Department of Cell Biology, Albert Einstein College of Medicine, New York, NY 10461, USA; 6 Institut Pasteur, Unité de Génétique des Mammifères, Paris, France; 7 Department of Immunology and Infectious Disease, The John Curtin School of Medical Research, the Australian National University, 131 Garran Road, Canberra 2601, Australian Capital Territory, Australia; 8 Department of Haematology, University Hospital, Nîmes. Faculty of Pharmacy and Research Team EA 2992, University of Montpellier, Montpellier, France; 9 GIG-EA 7404, Université de Versailles-Saint Quentin en Yvelines, Unité de Formation et de Recherche des Sciences de la Santé Simone Veil, 78180 Montigny-le-Bretonneux, France; 10 Service de Biologie Médicale, Centre Hospitalier de Poissy-Saint-Germain, 78300 Poissy, France; 11 Centre National de la Recherche Scientifique UMR 7278, IRD198, INSERM U1095, Aix-Marseille Université, Marseille, France; 12 Department of Medicine, Weill Medical College of Cornell University, New York, NY 10021, USA; 13 INRA–CNRS, Université de Tours—Haras Nationaux, IFR 135, 37380 Nouzilly, France

**Keywords:** recurrent spontaneous abortion, implantation, interspecific recombinant congenic mice

## Abstract

Recurrent spontaneous abortion (RSA) is a common cause of infertility, but previous attempts at identifying RSA causative genes have been relatively unsuccessful. Such failure to describe RSA aetiological genes might be explained by the fact that reproductive phenotypes should be considered as quantitative traits resulting from the intricate interaction of numerous genetic, epigenetic and environmental factors. Here, we studied an interspecific recombinant congenic strain (IRCS) of *Mus musculus* from the C57BL6/J strain of mice harbouring an approximate 5 Mb DNA fragment from chromosome 13 from *Mus spretus* mice (66H-MMU13 strain), with a high rate of embryonic resorption (ER). Transcriptome analyses of endometrial and placental tissues from these mice showed a deregulation of many genes associated with the coagulation and inflammatory response pathways. Bioinformatics approaches led us to select *Foxd1* as a candidate gene potentially related to ER and RSA. Sequencing analysis of *Foxd1* in the 66H-MMU13 strain, and in 556 women affected by RSA and 271 controls revealed non-synonymous sequence variants. *In vitro* assays revealed that some led to perturbations in FOXD1 transactivation properties on promoters of genes having key roles during implantation/placentation, suggesting a role of this gene in mammalian implantation processes.

## 1. Introduction

Human infertility represents a public health concern affecting 10–15% of all couples [1]. Despite advances in diagnosis and treatment, approximately 30% of cases are still considered idiopathic [2]. Recurrent spontaneous abortion (RSA) is clinically defined by at least three pregnancy losses prior to the 20th week of gestation and is a common cause of infertility, because it affects 1% of all pregnancies [3–6]. Approximately 50% of those are considered idiopathic, thereby underlining the disease's potential genetic and epigenetic causes. Unfortunately, previous attempts at identifying RSA causative genes have been relatively unsuccessful. Several candidate genes (e.g. *AMN*, *TM*, *EPCR*, *VEGF*, *p53*, *eNOS*, *JAK2*, *MTHFR*, *WNT6*) have been studied, especially using Sanger sequencing, but only a few variants and genotypes have been associated with the phenotype [7–11]. Although some genetic markers indicative of an elevated risk of being affected by RSA have been proposed, functional evidence is rare. This has restricted their efficient use in clinical studies. Genome-wide scan-based studies have been reported, although they have not reached the classical accepted statistical threshold for significance and have apparently failed to identify specific genes [12,13]. Such failure to identify RSA aetiological genes might be explained by the fact that reproduction's inherent complexity theoretically implies that mutations in hundreds of candidate genes may be responsible for the phenotype [10,14]. Furthermore, the genetic study (e.g. via classical genetic linkage analysis or GWAS) of families affected by RSA is particularly challenging owing to their rarity. Indeed, causative variants related to reproductive fitness are under strong negative selection. Compared with genetic analysis of ovarian infertility, which allowed discovery of interesting genes [15,16], pertinent candidates genes were seldom found when the infertility was linked to placental/endometrial defects, such as RSA [14].

It is worth stressing that mammalian reproductive phenotypes (and their inherent molecular mechanisms) should be considered as quantitative traits resulting from the intricate interaction of numerous genetic, epigenetic and environmental factors. With that in mind, our group took advantage of a particular mouse interspecific recombinant congenic strains (IRCS) model allowing the identification of quantitative trait loci (QTL) related to complex phenotypes [17–21]. Phenotyping of IRCS animals enabled us to map several QTL related to embryonic resorption (ER) and lethality [20,22]. Murine ER might be caused by molecular disturbances regarding implant and fetal–placental unit function, and it is highly plausible that causal genes play similar roles in human diseases, such as RSA, preeclampsia and/or fetal growth restriction [23–25].

In a previous study, we showed that the 66H-MMU13 IRCS strain is affected by a high ER rate (14.7% versus 4.6% observed in C57BL/6 J females, *p* < 0.01). This strain contains a unique approximately 5 Mb *spretus* fragment on chromosome MMU13 (between rs120693734 and D13Mit47 polymorphic markers) encompassing 31 genes [22].

This study includes whole transcriptome analysis of endometrial and placental tissues from the IRCS 66H-MMU13 presenting with a high ER rate. Transcriptomics and bioinformatics approaches led us to select *Foxd1* (a forkhead transcription factor located in the critical fragment) as a candidate gene potentially related to ER and RSA. Sequencing analysis of *Foxd1* coding regions in the 66H-MMU13 strain, *Mus spretus* animals, 556 women affected by RSA and in a control population revealed many non-synonymous sequence variants. *In vitro* assays revealed that some of them (e.g. *Foxd1*-p.Thr152Ala, FOXD1-Ala356Gly, FOXD1-Ile364Met and FOXD1-429AlaAla) had a functional effect as they led to perturbations in FOXD1 transactivation properties on promoters of the Placental Growth Factor (*PGF*) and the complement component gene (*C3*) having key roles during implantation/placentation [26–28]. Finally, with our study, we found that women with *FOXD1* mutations have a statistically high risk (10.3 relative risk) of suffering RSA.

Taken together, our results showed that *FOXD1* is a major actor in mammalian reproduction as sequence variants generated ER and RSA in mice and humans, respectively. We propose that the FOXD1-p.429AlaAla mutation might be used as an RSA molecular marker while FOXD1 p.Ala88Gly variant might have a protective effect.

## 2. Material and methods

### 2.1. Expression microarrays from uterine and placental mouse tissues

Eight 66H-MMU13 females were crossed with C57BL6/J males, following a previously described mating protocol [22]. Female mice were euthanized by cervical dislocation at E12.5, in accordance with Paris Descartes University, the Cochin Institute and the Guidelines for Biomedical Research Involving Animals policies (no. 13-020: ‘Implication of FOXD1 in ER and RSA’ reference no. 00175.01). Placentas were dissected from live embryos and total RNA extracted using Trizol. Total RNA was also extracted from uterine tissue located between contiguously implanted (normal development) embryos. A pool of four placentas and four uteri from each mouse was used. Four micrograms from each RNA pool were sent to a NimbleGen expression array platform for DNA end-labelling, hybridization, scanning and data normalization, thereby providing the final data file. The data are available from Annotare 2.0 (accession number: E-MTAB-4643).

### 2.2. Bioinformatics analysis of microarray data

Placenta and uterus were compared between C57BL6/J mice and 66H-MMU13 mice, using the GSEA tool (http://software.broadinstitute.org/gsea/index.jsp), in order to identify the most relevant gene sets, first against the hallmark dataset collection, and then against the c2.all.v5.1.symbol.gmt dataset. The hallmark dataset encompasses only 50 gene sets with very clear characteristics summarizing large biological functions. The other dataset encompasses 4276 gene sets and allows a much more detailed analysis of the transcriptome.

### 2.3. Bioinformatics and biostatistics

Statistical analysis (Student's *t*-test, followed by Bonferroni correction for multiple testing) identified putative transcription factor binding sites (TFBS) using the Genomatix Gene2Promoter option. Putative TFBS in the promoters of the 50 most upregulated genes were compared with the 40 most downregulated ones for such analysis. Student's *t*-test was used for assessing binding site statistical representation by comparing their occurrence in two groups of promoters for each putative binding site identified. The *t*-test was corrected to take multiple testing into account. The putative binding sites identified by promoter were classified according to their degree of discrepancy (in terms of frequency) between both groups of promoters. Electronic supplementary material, table S1 outlines this analysis for Forkhead Binding sites that were the most significantly enriched in promoters of down-regulated genes in the microarray experiment. SIFT (http://blocks.fhcrc.org/sift/SIFT.html), PolyPhen-2 (http://genetics.bwh.harvard.edu/pph2/, Mutation Taster (http://www.mutationtaster.org/) and Align GVGD (http://agvgd.hci.utah.edu/) software, as well as data from the 1000 genome database (electronic supplementary material, table S3), were used for determining the potential deleterious effect of amino acid changes [29–33].

### 2.4. *Foxd1* sequence analysis in mice

The complete *Foxd1* coding sequence*,* as well as 5′ and 3′ flanking regions, were amplified in two separate amplicons (named fragment 1 and 2), using Kapa HiFi polymerase (Clinisciences) following the manufacturer's protocol. Electronic supplementary material, table S2 lists the oligonucleotide sequences. PCR conditions were identical for both fragments. PCR products were treated with shrimp alkaline phosphatase and exonuclease I, following the manufacturer's recommendations (USB). The following primers were used for the mouse sequence reaction: mfoxd1-1F, mfoxd1-1R and mfoxd1-2F, mfoxd1-2R for fragments 1 and 2, respectively. Sequence analysis was completed at the Cochin Institute's Genomics Platform, using an ABI 3100 sequencer (Applied Biosystems, Foster City, CA, USA).

### 2.5. Human *FOXD1* sequence analysis

The entire *FOXD1* coding region was amplified in two separate amplicons (HuFrag-1 and HuFrag-2) or as a unique amplicon using KAPA hifi polymerase (Cliniscience) with the primers presented in the electronic supplementary material, table S2. PCR products were treated with shrimp alkaline phosphatase and exonuclease I, as described by the manufacturer (USB). Sequence analysis involved using the following oligonucleotides: HuFOXD1-1Fb and HuFOXD1-174F (for HuFrag1), HuFOXD1-2R2b, HuFOXD1-OM and HuFOXD1-980R (for HuFrag2) using an ABI 3100 sequencer (Applied Biosystems). All non-synonymous variants found in the RSA and control group were confirmed by novel sequence analysis from new *FOXD1* PCR products.

### 2.6. Plasmid constructs: *FOXD1* expression vectors

The mouse *Foxd1* coding sequences (mutant: Thr152Ala, WT *musculus* or *spretus*) were introduced into the pcDNA3.1/CT-GFP topoTA cloning vector (Invitrogen, Carlsbad, CA, USA). All expression constructs were sequenced to confirm the presence of the expected variants and to exclude PCR-induced mutations. The human *FOXD1* coding sequences from patients (carrying the FOXD1-Ala356Gly, FOXD1-Ile364Met and FOXD1-429AlaAla mutations) as well as from a WT individual were amplified using the HuFOXD1-a and HuFOXD1-OM oligonucleotides. Purified PCR products were cloned into the pcDNA3.1/CT-GFP topoTA cloning vector (Invitrogen).

### 2.7. Plasmid constructs: promoter reporter vectors

The murine *Pgf* promoter consisted of 2066 bp, encompassing the −2300 to −235 bp region upstream of the ATG initial start codon. The *C3* promoter region consisted of 810 bp (from −811 to −2 bp upstream of the initial ATG start codon). Each amplicon was generated using 250 ng DNA and Platinum Pfx DNA polymerase (Invitrogen). Amplicons were introduced into the pCR4-topoTA cloning vector (Invitrogen) and directly sequenced after purification with shrimp alkaline phosphatase and exonuclease I. All expression constructs were sequenced to confirm the presence of the expected variants and to discard PCR-induced artefacts. These fragments were purified and cloned, using T4 DNA ligase (Invitrogen), into a previously digested PGL3-basic luciferase vector (Promega).

The human *PGF* promoter amplicon consisted of 720 bp (−1144 to −424 bp upstream of the initial ATG start codon) or 1653 bp upstream of the ATG start codon for the long version of the promoter. The human *C3* promoter amplicon consisted of 729 bp (−792 to −63 bp upstream of the initial ATG start codon). These fragments were amplified from genomic DNA from a control individual having non-mutant sequences and then compared to AC_000146.1 and NC_000019.8 (NCBI). Amplicons were introduced into the pCR4-topoTA cloning vector (Invitrogen) and sequenced to exclude PCR-induced mutations. The *PGF* and *C3* promoter regions were then extracted. These fragments were purified and cloned, using T4 DNA ligase (Invitrogen), into a previously digested PGL3-basic luciferase vector (Promega).

### 2.8. Cell culture and luciferase assays

The calcium phosphate method was used for co-transfecting either COS-7 or KGN cells with 400 ng of any *FOXD1* construct and 570 ng of any PGL3 plasmid containing the target genes' promoter regions (COS-7 cells were used due to their high capacity of being transfected, and used as a classical cell model; KGN are ovary cells, whose expression profile may be closer to the reproductive expression characteristics of the female genetic tract). Experimental controls consisting of PGL3-basic and pcDNA3.1/CT-GFP empty vectors were included for each condition. The cell medium was replaced by a fresh one 28 h after transfection. The FOXD1 mutants' transcriptional activity was assessed 44 h after transfection using the Dual-Luciferase Reporter Assay System (Promega, Madison, WI, USA). Each experiment was performed at least twice in sixplicates. The firefly activity observed for each replicate was divided by the activity recorded for the Renilla luciferase vector. Student's *t*-test was used for estimating statistical significance. Transfection with empty pcDNA expression vector was used to normalize a potential effect on each promoter. Statistixl add-on software for Excel was used for statistical analysis (ANOVA), followed by post hoc tests (Student–Newmann–Keuls).

### 2.9. Patients and controls

The RSA patient group consisted of 556 women who had enrolled in a matched case-control study of unexplained pregnancy loss in the Nimes Obstetricians and Haematologists (*n* = 2175 patients) [26]. The control group consisted of 271 women who had at least one live birth with no history of pregnancy loss. All these subjects (RSA patients and controls) were Caucasian.

## 3. Results

### 3.1. Transcriptomics and gene ontology analyses reveal downregulation of the coagulation cascade and the inflammatory response in placenta and uterus of 66H-MMU13 strain

**Figure 1.**
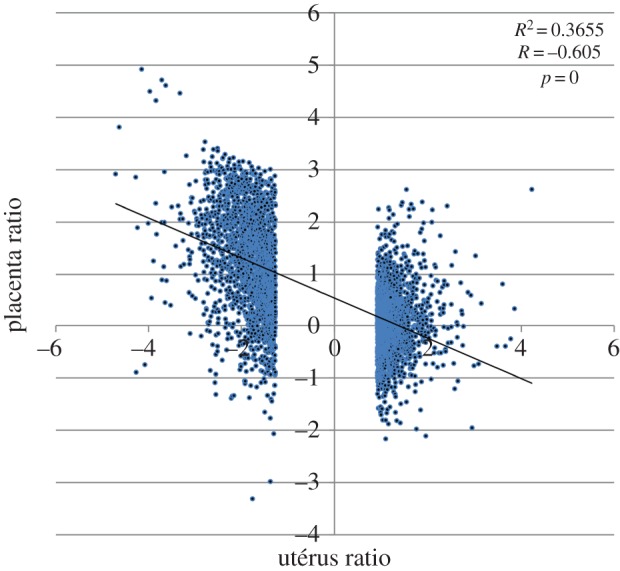
Induction ratios in the uterus and the placentas of mice between WT and 66H-MMU13, showing clearly that there is an inverse correlation of the alterations between the placenta and the uterus.

To investigate the mechanism underlying the ER phenotype observed in the 66H-MMU13 strain, we undertook a comparative analysis of endometrial and placental tissues (comparing 66H-MMU13 versus C57/BL6 animals, which differ only by an approx. 5 Mb DNA fragment located on chromosome 13 that is of *Mus spretus* origin in the 66H-MMU13). This analysis revealed important gene expression deregulation (electronic supplementary material, table S1). Analysis of 66H-MMU13 and C57BL6/J uterine tissue revealed that 6.8% and 4.3% of genes are up- and downregulated, respectively, at the threshold of twofold (compared with *Mus musculus* levels) while in placental tissue, 3.6% and 7.2% of genes were up- and downregulated, respectively. Finally, 2.3% of genes were simultaneously and systematically upregulated in the uterus and downregulated in the placenta (‘mirror effect’), as presented in figure 1, showing a negative correlation estimated at *r* = −0.605 (*p* < 10^−300^), between the deregulation of genes in the placenta versus the deregulation in the uterus.

**Figure 2.**
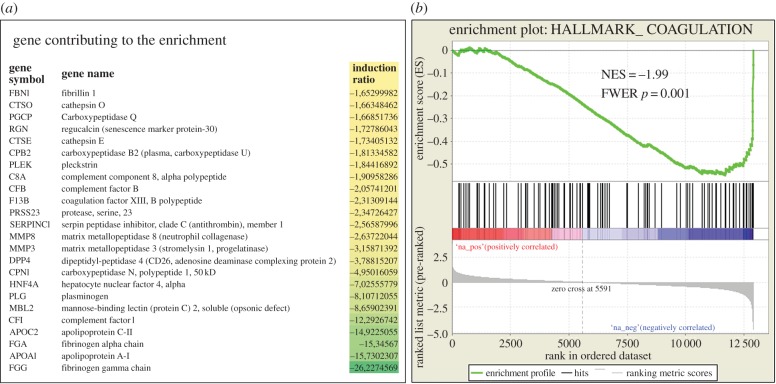
The major gene set of downregulated gene in the placenta is composed of genes involved in the regulation of coagulation. (*a*) The list of the most strongly downregulated in this pathway, from the GSEA analysis (presented in part (*b*) of the figure). NES reflects the Normalized Enrichment Score, indicating a very significant enrichment of genes involved in this pathway.

We then used GSEA to identify gene sets significantly enriched among the genes found deregulated in the placentas and uteri of 66H-MMU13 versus C57/BL6. First, comparing the reference hallmark gene set with the placental deregulated genes, we could identify two gene sets out of 50 that were significant with an FWER < 0.05 in the upregulated genes (WNT_BETA_CATENIN_SIGNALING and _APICAL_SURFACE), and two gene sets in the downregulated genes (COAGULATION and OXIDATIVE_PHOSPHORYLATION). The most significant by far was the ‘Coagulation’ hallmark (FWER *p*-value = 0.001) as presented in figure 2. Second, the C2 reference gene symbol set was used as reference (this group containing 4726 gene sets). Four gene sets were upregulated with an FWER < 0.05 (MARTENS_TRETINOIN_ RESPONSE_UP, MIKKELSEN_MCV6_HCP_WITH_H3K27ME3, KEGG_BASAL_CELL_CARCINOMA and MEISSNER_BRAIN_HCP_WITH_H3K27ME3), whereas 20 gene sets were downregulated at the same threshold (table 1). Among those, many groups of genes involved in liver function were found, especially in link with coagulation and targets of Hepatocyte Nuclear Factors, HNF4α, HNF3β or HNF1α. These factors do not share common sequences but are all of great importance in the liver. They are also involved in coagulation as well as fibrin clotting cascades. Other groups of genes included steroid hormone biosynthesis, lipid transport and lipoprotein metabolism.

**Table 1.** Highly significant placental gene sets identified as enriched between 66H-MMU13 and C57B6/J mice. The gene sets (obtained from GSEA, http://software.broadinstitute.org/gsea/msigdb/collections.jsp) were systematically tested against the placental transcriptome comparing the two mouse lines under scrutiny. When a name starts the geneset it refers to the researcher that published the dataset. Size refers to the number of genes present in the geneset. Positive values for ES and NES (light grey) stands for gene clusters of upregulated genes, while the negative values (dark grey) account for clusters of downregulated genes. NOM p-value is the nominal (non-corrected *p*-value). FDR is the false discovery rate and FWER is the family-wise error rate. The RANK at MAX refers to the position of the last gene inside the up- or downregulated group, among the complete classified list of genes from the microarray experiment. For instance, this means that statistically there are 2.45-fold more genes than expected in the present study that are correlated to upregulated genes following tretinoin treatment.

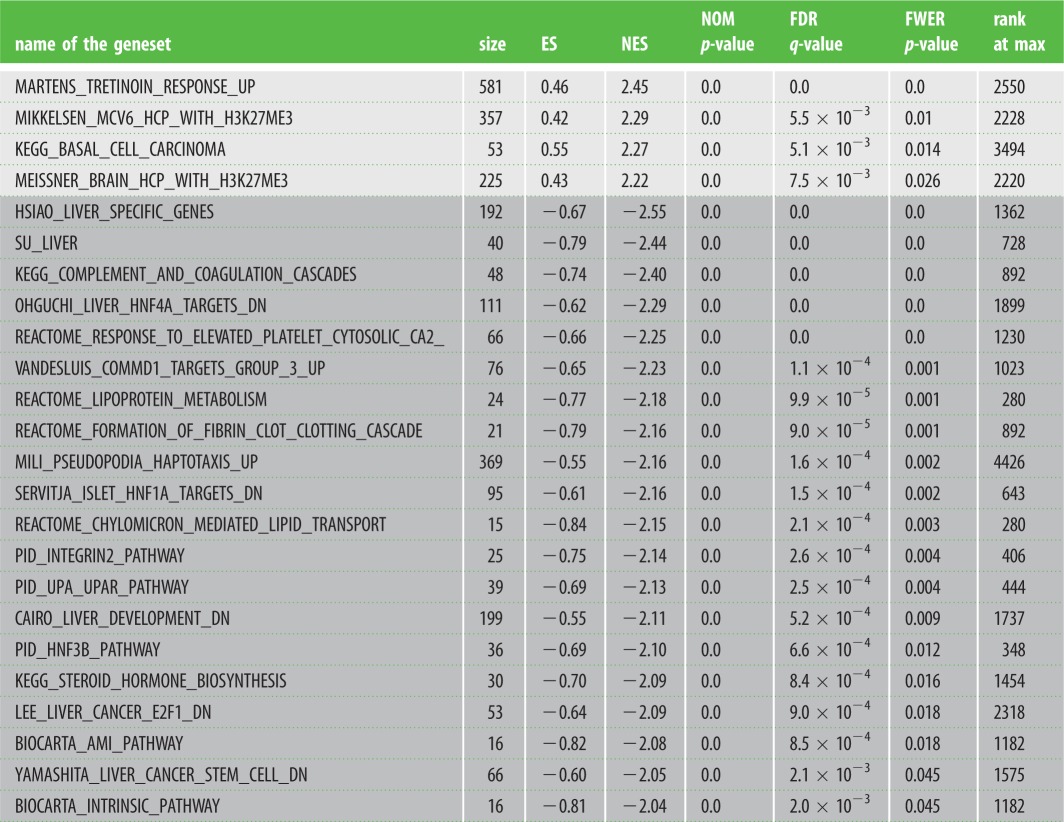

**Figure 3.**
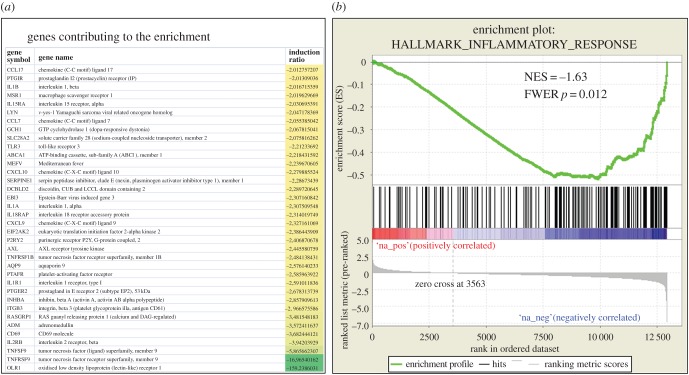
The major gene set of downregulated genes in the uterus is composed of genes involved in inflammation. (*a*) The list of the most strongly downregulated genes in this pathway, from the GSEA analysis (presented in part (*b*) of the figure). NES reflects the Normalized Enrichment Score, indicating a very significant enrichment of genes involved in this pathway.

We performed a similar GSEA analysis on genes found deregulated in uteri of 66H-MMU13 versus C57/BL6. Only one hallmark entitled ‘PANCREAS_BETA_CELLS’ was found significant in the upregulated genes list (FWER = 0.008), suggesting that alterations of uterine gene expression resemble those found in the pancreas (the genes involved in the detection of this cluster were *Insm1*, *Chga*, *Isl1*, *Pklr*, *Sst*, *G6pc2*, *Pcsk2*, *Neurod1* and *Nkx6-1*, which induced 5.1-, 2.3-, 2.1-, 1.7-, 1.6-, 1.5-, 1.4-, 1.3- and 1.25-fold, respectively). By contrast, six hallmarks were significant in downregulated genes, which are INFLAMMATORY_RESPONSE, INTERFERON_GAMMA_RESPONSE, TNFA_SIGNALING_VIA_NFKB, IL2_STAT5_SIGNALING, IL6_JAK_STAT3_SIGNALING and ALLOGRAFT_REJECTION. The first of them is represented in figure 3 along with the most downregulated genes. On the whole, the uterus of the 66H-MMU13 mice was characterized by a strong downregulation of genes involved in inflammation and immunity. The analysis of the C2 gene set from GSEA confirmed a great contrast between the number of upregulated gene sets (48 with an FDR < 0.25) and downregulated gene sets (711 gene sets with a FDR < 0.25). In the downregulated clusters, a vast quantity of inflammatory/immune pathways are present (related to viral infection, IFN targets, CTLA4 pathway, Graft-versus host disease, etc.). In the upregulated groups, beta cell development is present, consistent with the hallmarks previously identified.

### 3.2. Promoter analysis of deregulated genes identifies *Foxd1* as the most relevant candidate gene to explain 66H-MMU13 phenotype

The list of genes present in the *Mus spretus* fragment has previously been published [22]. Several genes in this region are putatively involved in the phenotype observed, such as F2rl1, F2r. Bioinformatics analysis of putative TFBS using Genomatix showed that transcription factors (TFs) encompassing forkhead binding sites (FKHD) in placental tissue were at the top of the list (statistical discrepancy between promoters of repressed and induced genes). More specifically, repressed genes contained twice as many FKHD than promoters of induced genes (2.54 versus 1.27, *p* = 0.0009; electronic supplementary material, table S1 and figure S1). The fragment of *spretus* chromosome 13 fixed in the 66H-MMU13 strain contains only one forkhead domain transcription factor: *Foxd1*. It is therefore a strong candidate to explain the gene deregulation associated with the elevated ER in 66H-MMU13 line. Because *Foxd1* was not modified in the array, our hypothesis was that the *spretus* and *musculus* versions of *Foxd1* had different effects on gene regulation, rather than one version being differentially expressed compared with the other.

### 3.3. *Spretus* version of *Foxd1* presents sequence variants which have functional consequences on its target genes

**Figure 4.**
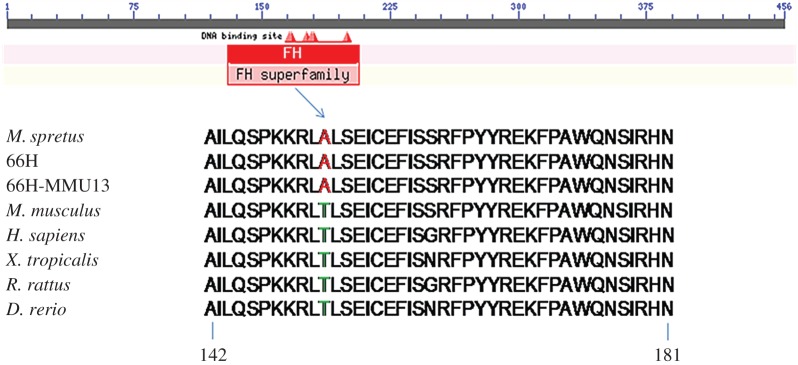
Alignments of FOXD1 DBD in different species of vertebrates. Note the difference between the spretus-derived samples and all the other species at position 152.

Direct sequencing of the complete *Foxd1* coding region from *Mus spretus* and 66H-MMU13 animals revealed five non-synonymous variants relative to the C57B6/J version: p.Asp73Glu, p.Asn126Glu, p.Thr152Ala, p.Asp76_Leu77InsAsp and p.Pro319del. One specific variant, Foxd1-Thr152Ala, drew our attention because it is located in the protein's forkhead DNA-binding domain (DBD) and threonine in position 152 is strictly conserved during evolution except in *spretus* (figure 4). The SIFT software predicted a highly deleterious effect of a change of this threonine into an alanine (as found in *spretus*; score: 0.00) as this mutation triggers the disappearance of a predicted phosphorylation hot-spot. We next tested the functional effect of p.Thr152Ala variant on promoters of known *Foxd1* target genes which were among the deregulated genes in microarray analyses of 66H-MMU13 placenta/uterus: *Pgf* and *C3*.

**Figure 5.**
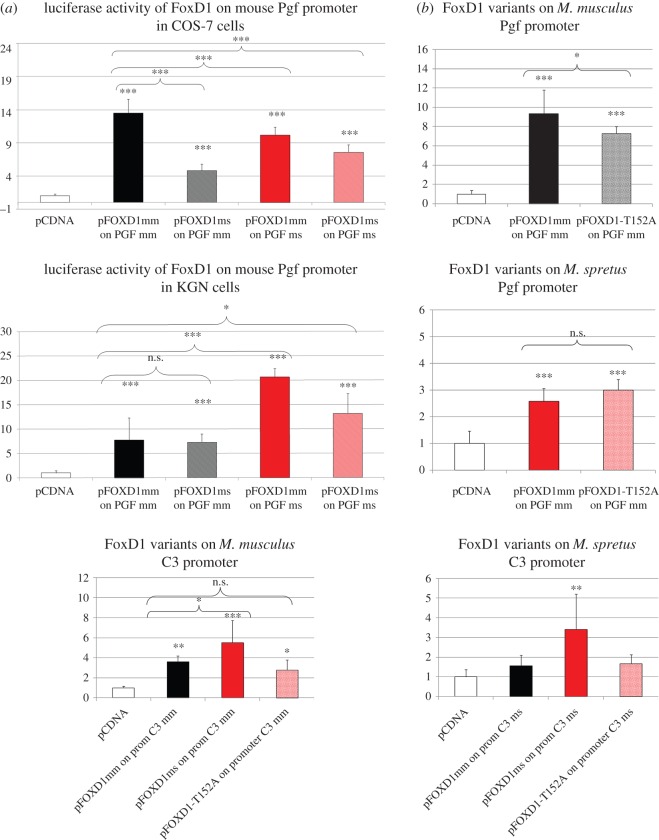
Transactivation properties of distinct mouse Foxd1 versions on Pgf and C3 promoters. (*a*) Luciferase activity on the mouse Pgf promoter in two cell models (COS-7 and KGN). In black are the effects of the *Mus musculus* and *Mus spretus* versions of Foxd1 on the *Mus musculus* promoter. In red are the effects of the same TFs on the *Mus spretus* promoter. The expression profiles are similar, despite significant differences in the cell lines. Overall, the activation of the promoter appears more efficient with the *Mus musculus* FoxD1. (*b*) The effects of specific variants of FoxD1, including the T152A mutation on the Pgf and C3 promoters. The *Mus spretus* variant appears more efficient on the C3 promoter and less efficient on the *Mus musculus* version of the Pgf promoter. These results are consistent with an overexpression of complement cascades (negative for implantation) and underexpression of a major actor of placental angiogenesis, Pgf. *p*-values *<0.05, **<0.01 and ***<0.001.

In luciferase assays, the *spretus* version of Foxd1 (Foxd1-ms) was able to transactivate *Mus musculus Pgf* (*Pgf*-mm) promoter but to a lesser extent (approx. threefold reduction, *p*-value < 0.001) than Foxd1 *Mus musculus* version (Foxd1-mm). Similarly, Foxd1-mm transactivation of the *spretus Pgf* (*Pgf*-ms) promoter was reduced compared with its effects on *Pgf*-mm promoter. However, Foxd1-mm transactivation of *Pgf*-ms promoter was significantly higher than that of *spretus* Foxd1 version (Foxd1-ms), suggesting that Foxd1 *Mus musculus* version leads to a higher activation of Pgf promoter whether from *musculus* or *spretus* (figure 5). The Foxd1-Thr152Ala mutation alone significantly decreased Foxd1 ability to transactivate *Pgf* promoter. Conversely, Foxd1-ms overexpression led to higher *C3*-mm promoter transcription activity than that induced by the Foxd1-mm version while the Foxd1-T152A mutation alone had no significant differences regarding control (figure 5). On the whole, these data demonstrate that Foxd1 *spretus* and *musculus* versions have different effects on their target genes, in agreement with a model in which the gene deregulation and associated ER phenotype in 66H-MMU13 strain are mainly due to sequence variants in *Foxd1 spretus* version, compared with *musculus*.

### 3.4. *FOXD1* sequencing in humans show polymorphisms with effects associated with adverse pregnancy outcomes

As the results we obtained in mice were encouraging, we move to human patients and sequenced *FOXD1* in 556 women affected by RSA and 271 women with normal fertility, and found a total of 27 sequence variants (table 2). Nine of them were present in both RSA and control individuals, 18 (10 non-synonymous) variants were identified only in RSA patients and one was present exclusively in control individuals. Sequence variants found in RSA women concerned 33 patients (5.9%). All non-synonymous nucleotide changes exclusive to the RSA group (not present in control individuals) were identified in heterozygous state. A contingency *χ*^2^-test revealed highly significant (*p* = 0.0006) statistical comparison of the exclusive variants in the patient population to those in the control group. Relative risk calculated using all these ‘private’ mutations was 10.3 (5% confidence interval: [1.4–77.2]). The statistical significance was achieved using a single sequence variant (p.429AlaAla) in which two alanine residues were inserted at protein position 429. This mutation was found 12 times exclusively in the patient group (2.2%, *p* = 0.015). The p.Ala88Gly variant was found in 8.5% of the women from the control group (3.6% in the RSA group).

**Table 2.** *FOXD1* open reading frame sequencing in RSA patients and control individuals. Mutations tested for their functional impact are in bold.

DNA	protein	patients (*n*=556)	controls (*n*=271)	*p*-values < 0.05
c.69G>C	p.Gly23Gly	7	3	
c.237G>A	p.Leu79Leu	3	0	
c.300C>T	p.Ala100Ala	1	0	
c.324 C>T	p.Gly108Gly	2	0	
c.612G>A	p.Glu204Glu	1	2	
c.903C>A	p.Ala301Ala	1	0	
c.1007 C>T	p.Ala336Val	1	0	
c.1248G>C	p.Val416Val	1	0	
c.1297 GCC>GCG	p.Ala432Ala	2	0	
c.1308A>G	p.Ser436Ser	318	136	
c.1055 C>G	p.Arg352Pro	1	0	
c.1007 C>T	p.Ala336Val	1	0	
c.164G>C	p.Arg55Pro	1	0	
c.263G>C	p.Ala88Gly	20	23	0.004
c.326_327InsGCG	p.Ins109Gly	1	0	
c.683C>T	p.Pro228Leu	0	2	0.043
c.721G>C	p.Ala241Pro	1	0	
c.976G>A	p.Ala326Thr	21	8	
**c.1067 C>G**	**p.Ala356Gly**	**1**	**0**	
**c.1092 C>G**	**p.Ile364Met**	**2**	**0**	
c.909_1165del256	p.FS>STOP376	1	0	
c.1146-1160del	p.Gln383_Ala387del	27	13	
c.1169_1170InsGGCCGC	p.Ins391ProPro	6	7	
c.1187C>T	p.Pro396Leu	3	1	
**c.1285_1286InsGCCGCG**	**p.Ins429AlaAla**	**12**	**0**	**0.016**
c.1309G>A	p.Val437Ile	1	0	
c.1324G>T	p.Ala442Ser	1	0	

**Figure 6.**
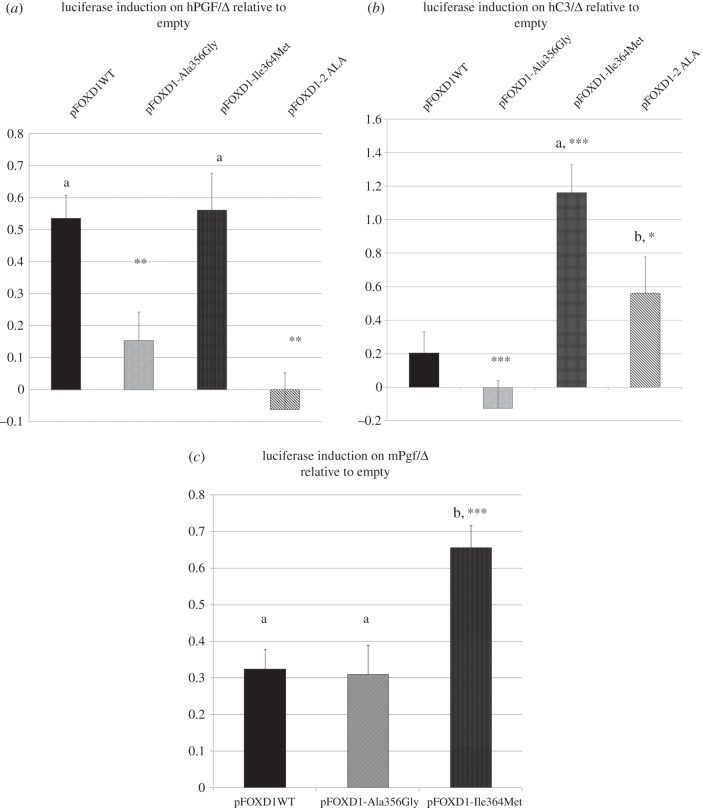
Transactivation properties of human WT and mutant FOXD1 versions on human PGF and C3 promoters. (*a*,*b*) The two mutants found in RPl patients Ala356Gly pFOXD1-2ALA are unable to activate the PGF promoter efficiently, while the A706 and 2-ALA variants trigger overexpression of C3 (significant only for A706: p.Ile364Met). The A706 mutant seems to be able to provoke an overexpression of the mouse Pgf promoter (*c*). Letters reflect significant differences compared with transfections with the empty expression vector. Asterisks relate to the comparison relative to the WT induction: *p*-values *<0.05, **<0.01 and ***<0.001, compared to WT. Error bars represent standard error.

In luciferase assays, FOXD1-p.Ala356Gly and FOXD1-p.429AlaAla forms were completely unable to activate the *PGF* promoter while the FOXD1-Ile364Met variant retained a transactivation capacity similar to that of the WT form. Regarding the effect on *C*3 promoter activity, the FOXD1-Ala356Gly versions did not yield to any activation of *C3* promoter, contrary to the WT form, while the Ile364Met form induced an approximately sixfold transactivation. The FOXD1-429AlaAla variant also tended to induce the expression level (approx. threefold) of *C3* promoter than the WT form (figure 6).

## 4. Discussion

Human fertility, like most biological processes in mammals, is assumed to be the result of subtle interaction of gene variants located in different genomic regions, having a quantitative effect and thus called QTL for quantitative trait loci. The expression of these gene variants, in interaction with environmental factors, results in significant quantitative phenotypic differences between individuals (e.g. at the organ or at the molecule levels). Classical genetic approaches (e.g. genetic linkage analysis in families, direct sequencing of candidate genes) have been relatively unsuccessful for discovering genes modulating fertility in the endometrial/placental context, probably because in this case the failure results in a very complex interaction between two genetically different partners (the placenta with half of the alleles coming from the father, on the one hand, and the uterus, on the other hand), involving highly complex and strongly regulated immune tolerance mechanisms. The genetics of infertility QTL was often studied in mouse models, leading for instance to identification of regions of the X chromosome implied in male infertility in mice [34,35]. However, these approaches have generally not permitted gene identification [22]. Similarly, in species where the economic importance of controlling fertility is huge, such as in cattle, genomic regions containing genes involved in fertility have been identified as well, but rarely genes [36].

The IRCS model that we used in this study presents some advantages such as the high genetic and phenotypic variability between parental strains (*Mus musculus* and *Mus spretus*). Because the average length of the *spretus* chromosomal fragments fixed in the *Mus musculus* genetic background is small, it allows a relatively precise identification of the QTL location, albeit the ultimate cloning of relevant genes remains very challenging [37].

A previous study showed that the 66H-MMU13 IRCS strain was affected by a high ER rate (14.7% versus 4.6% observed in C57BL/6 J females, *p* < 0.01). This strain contains a unique 5 Mb *spretus* fragment on chromosome MMU13 (between rs120693734 and D13Mit47 polymorphic markers) encompassing 31 genes [22]. In this study, the classical genetic approaches are combined with cDNA microarray assays in relevant tissues (endometrium and placenta) for evaluating potential placental and uterine transcriptional differences between 66H-MMU13 and control animals that could explain the phenotype. This hypothesis proved reliable because it has been previously shown that incompatibilities between separate chromosome regions in the IRCS model induce strong gene expression alterations, due to interspecific genomic divergence between coding (e.g. TFs) and regulatory regions (e.g. promoters). Indeed, in the IRCS mice, two genomes of two different species that diverged around 1 Ma are collapsed in a few generations, leading to a phenomenon named ‘transcriptomic shock’ [37,38].

Comparative analysis of endometrial and placental tissues' transcriptomes between 66H-MMU13 and C57BL/6 J animals showed deregulation of gene expression throughout the whole genome. Some genes show systematic opposite deregulation (‘mirror effect’) between placenta and uterus, suggesting an alteration of the bidirectional molecular dialogue normally occurring between these tissues. Such widespread modification in expression profiles might be due to alterations of one or several TFs located on the 66H-MMU13 strain's *spretus* fragment. Following such an assumption, a *Mus spretus*-TF might have abnormal transactivation properties on multiple *Mus musculus* target promoter sequences. Bioinformatics analysis of the promoter content in TF-binding sites in the deregulated genes led us to propose *Foxd1* as a strong candidate. Direct sequencing of 66H-MMU13 and *spretus* animals demonstrated that, during evolution, five sequence variants were fixed in *Foxd1* coding regions of *Mus spretus* subspecies. *In silico* analysis underlined the strong interest of one of them (Foxd1-Thr152Ala), because it affected the forkhead DBD in a highly conserved position among mammalian species and the bioinformatics tools predicted a functional effect (figure 4).

A luciferase reporter assay was then carried out to investigate target gene transactivation capacity regarding both *Foxd1 spretus* alleles and the Thr152Ala variant, using two genes' promoter constructs (placenta growth factor, *Pgf* and complement component, *C3*). These were strongly modified in the microarrays and are known to play key roles in mammalian implantation [26,27,39,40]. The *Pgf* transcript (*Pgf* is a previously validated Foxd1 direct target gene [28]) was 4.3-fold less abundant in the 66H-MMU13 uterus compared with the C57BL/6 J control, while *C3* was upregulated 5.7-fold in the 66H placenta compared to C57BL/6 J control.

Luciferase reporter assays using the mouse *Foxd1* coding sequence and target promoters (*C3* and *Pgf)* showed that even though Foxd1-ms was able to stimulate *Pgf*-mm (interspecific) expression, activation became strongly and significantly altered (approx. 3-fold reduction) compared with that for Foxd1-mm on the *Pgf*-mm promoter (intraspecific). These results corroborated their biological pattern as observed in transcriptome assays. Reciprocally, it was observed that Foxd1-mm transactivation capacity regarding the *Pgf*-ms promoter (interspecific) became reduced compared with its effects on the *Pgf*-mm promoter (intraspecific). This could have been due to natural genomic polymorphism between *musculus* and *spretus* genomes, observed in terms of nucleotide substitution [38]. Interestingly, our experiments showed that the Foxd1-mm version was significantly more efficient in transactivating the *Pgf*-ms promoter than the Foxd1-ms version itself. Hypothetically, these features could be consistent with the idea that the *Mus spretus Foxd1* variants would modulate the amount of progeny to an optimal (and not maximal) number. On this point, it is worth noting that *spretus* litter size is naturally smaller than the *musculus* one (5.3 ± 1.4, versus approx. 7.6 in C57BL/6 J) [39,41]. We also tested the effect of the Foxd1-Thr152Ala mutation alone regarding *Pgf*-mm expression. As expected, this mutation significantly (albeit moderately) decreased Foxd1 transactivation capacity concerning the *Pgf* promoter, thereby contributing to downregulation of the *Pgf* levels observed in the 66H-MMU13 uterine tissue. Furthermore, it indicates that the other *Foxd1 spretus* variants also participated in this phenomenon (figure 5).

This led to investigating *spretus* Foxd1's functional impact on *Mus musculus* complement *C3*, a gene playing a central role in ER, as shown by the CBA×DBA cross [42]. Foxd1 *spretus* overexpression induced higher *C3*-mm expression levels than those induced by the Foxd1-*musculus* version (figure 5), while there were no significant differences between the isolated Foxd1-Thr152Ala mutation and control concerning this promoter. These *in vitro* experiments were consistent with the existing transcriptome data in which *C3* is 5.7-fold more abundant in the 66H-MMU13 placenta than in the C57BL/6 J tissue. Our experiments suggest that *C3* is a direct target of Foxd1, an observation which has not been reported before.

In sum, Foxd1 seems to regulate the expression of two crucial genes implicated in pregnancy maintenance. *Pgf* is highly expressed in the placenta, where it regulates vascular endothelial differentiation [43]. Uterine NK cells (uNK), an endometrial lymphocyte cell subset transiently found during endometrial decidualization and essential for immune dialogue with trophoblasts, express *Pgf* and *Vegf* [40,44,45]. *Pgf^−/−^* mice display defects during early differentiation of binucleate uNK cells [37]. Concerning *C3*, its activation is required for antiphospholipid-induced pregnancy loss that can be reverted by administering heparin, which blocks complement cascade activation [26]. The placenta appears to be subjected to a complement-mediated immune attack at the maternal–fetal interface during normal pregnancy. An appropriate complement inhibition is required for physiological gestation and, as has been thoroughly demonstrated in mice, the deficiency of complement regulatory proteins progressively leads to embryonic lethality [46,47]. Indeed, excessive local complement C3 production may overwhelm complement regulatory mechanisms, thereby leading to pregnancy loss. These findings suggest that strong *C3* expression disturbance (activation or inhibition) is related to embryo abortion.

Concerning *FOXD1* screening for mutations in women affected by RSA mutations, our statistical results showed that women having *FOXD1* mutations were at a high risk (10.3 relative risk) of suffering RSA. To the best of our knowledge, this value is the highest reported to date concerning idiopathic forms of RSA. The functional effects of three *FOXD1* mutations were assessed to establish a direct functional link between them and RSA aetiology. These mutations were predicted to be damaging, because they were exclusively found in RSA women, and most were not previously described in SNP databases, while their affected residues are conserved among mammalian species. The FOXD1 wild-type (WT) and mutant versions (FOXD1-Ala356Gly, FOXD1-Ile364Met and FOXD1-429AlaAla) were tested regarding their *PGF* and *C3* promoter transactivation properties (figure 6). FOXD1-Ala356Gly and FOXD1-429AlaAla were completely unable to activate the *PGF* promoter, while Ile364Met retained a transactivation capacity. These results suggested that FOXD1 variants may reduce PGF levels, thus potentially affecting pregnancy maintenance.

Concerning the *C3* promoter, the FOXD1-WT version induced *C3* compared with the empty vector while the FOXD1-Ala356Gly was unable to do so. FOXD1-Ile364Met versions induced significantly the C3 promoter, while there was a trend with the FOXD1-429AlaAla construct (not significant). Despite the non-signification due to interexperiment variation, *FOXD1* p.429AlaAla can be considered as hypomorphic and hypermorphic on *PGF* and *C3* promoters, respectively, both being problematic for a successful pregnancy.

It has been shown that C3 levels in humans are higher in patients suffering a third consecutive pregnancy loss than in women having a successful pregnancy after two idiopathic abortions [48]. Moderate *C3* expression levels in mice are necessary for successful gestation because *C3* knock-out (KO) animals have a much higher resorption rate 15 days *post coitum* than their WT counterparts, due to trophoblast dysfunction and labyrinth development defects [49]. Our *in vitro* experiments suggested that *FOXD1* mutations decreasing or increasing *C3* expression are deleterious. This indicates that a subtle tuning of *C3* expression levels is necessary for optimal fotal–placental interface function.

It should be noted that, up to now, severe defects have been seen in *Foxd1* KO animals regarding kidney (only in homozygous state) and optic chiasm development [50,51]. *FOXD1* human mutations or the mouse Foxd1-ms functional substitution described here displayed milder functional effects than those observed in *Foxd1* homozygous KO mice. This might be due to the mutation's intrinsic nature (complete KO versus point mutations) or/and to interspecific genetic/physiological differences.

This led to hypothesizing that more drastic mutations in humans may be related to kidney and/or neurological phenotypes. However, it cannot be ruled out that *FOXD1* could be implicated in syndromic forms of RSA which include these and/or other clinical features. It would be of interest to check for the presence of eye phenotype in patients suffering from RSA, which in these cases could be due to FOXD1 variants.

One concern in our work is the fact that the expression data about FOXD1 in the utero-placental unit is relatively scarce. The Protein Atlas database suggests, however, a significant expression of the protein in the human placenta (http://www.proteinatlas.org/ENSG00000251493-FOXD1/issue/placenta). The absence of reliable Foxd1 antibody in mice prevented us, despite several attempts, from observing a specific labelling, even in reference tissues such as the kidney. Furthermore, even the microarray data for this gene may sometimes be questionable due to the extreme richness in CG dinucleotides found in its open reading frame (73.8% versus 41% for the genome, with 264 CG and five *NotI* digestion sites (GCGGCCGC) in an Open Reading Frame of 1371 nucleotides while one is expected in more than 500 000 nucleotides on average), leading to possible problems in hybridization. It is interesting to note, however, that Sha and co-workers [52] published in 2007 a comparison of endothelial cells in the endometrium in endometriosis and control patients, which revealed a 6.1-fold increase (*p* = 0.0015) of FOXD1 mRNA level in the context of this disease, known to be a major cause of infertility. The expression levels reported in control endometrial endothelial cells from this study for FOXD1 corresponded to the expression of genes mildly expressed [52].

In conclusion, QTL positional cloning has been validated for the first time by genotyping an outbred species (human). The approach outlined here may be useful for other positional cloning projects where a limited set of recombination events is available for mapping purposes (as in IRCS, or more generally in mouse strains). Our results have also shown that *FOXD1* is possibly a new molecular actor modulating pregnancy maintenance, with mutations associated with ER in mice and RSA in humans. Our findings argue in favour of FOXD1 p.Ala88Gly conferring a protective effect, because it was frequently encountered in the control group. Finally, we propose that the FOXD1 p.429AlaAla mutation can be considered as an RSA molecular marker, which can be easily tested by PCR/sequencing.

## Supplementary Material

Figure S1

## Supplementary Material

Table S1

## Supplementary Material

Table S2

## Supplementary Material

Table S3
