# Supplementary figures and images for: Association of FOXD1 variants with adverse pregnancy outcomes in mice and humans
Source: Open Biol. 2016 Oct 19;6(10):160109. doi: 10.1098/rsob.160109 (PMC5090055; doi:10.1098/rsob.160109)

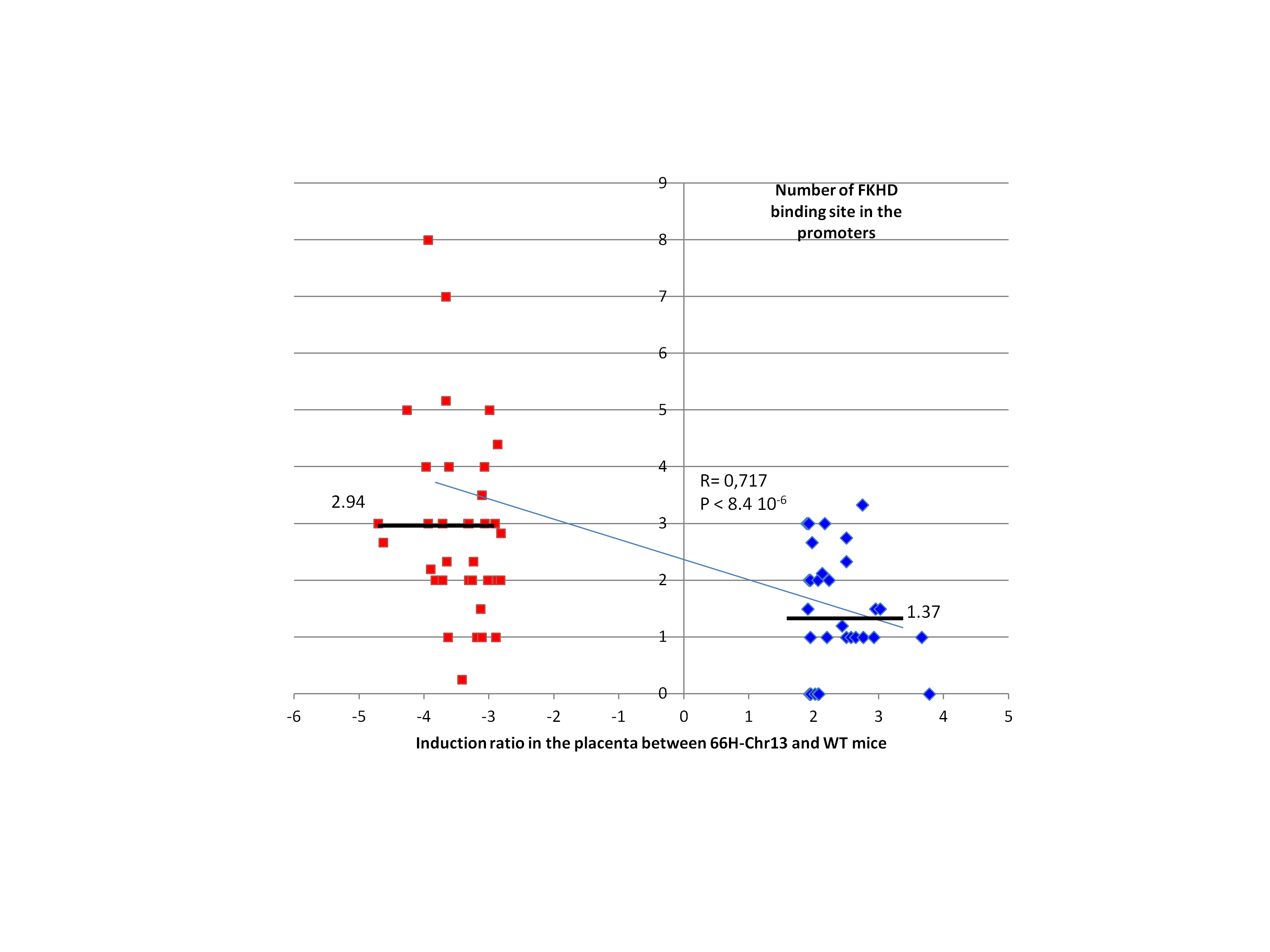

Supplement: Figure S1 [file rsob160109supp1.tif]
